# Valsalva Retinopathy Associated with Sexual Activity

**DOI:** 10.1155/2014/524286

**Published:** 2014-05-05

**Authors:** Khalid Al Rubaie, J. Fernando Arevalo

**Affiliations:** ^1^The Vitreoretinal Division, King Khaled Eye Specialist Hospital, Al-Oruba Street, P.O. Box 7191, Riyadh 11462, Saudi Arabia; ^2^Retina Division, Wilmer Eye Institute, Johns Hopkins University School of Medicine, Baltimore, MD 21231, USA

## Abstract

A 54-year-old healthy male presented complaining of sudden loss of vision in the right eye. Initial visual acuity was counting fingers. The patient's acute vision loss developed after sexual activity. Color fundus photos and fluorescein angiography were performed showing a large subinternal limiting membrane hemorrhage in the macular area. A 23-gauge sutureless pars plana vitrectomy with brilliant blue assisted internal limiting membrane peeling was performed with best-corrected visual acuity recovery to 20/50 at 6 months of followup.

## 1. Introduction

Valsalva retinopathy is an induced preretinal hemorrhage that can occur in the macular area. It usually occurs secondary to Valsalva's maneuver, a forcible exhalation effort against a closed glottis, causing a sudden rise in intrathoracic pressure. This unexpected increase in the venous pressure can rupture the perimacular vessels resulting in premacular hemorrhage [1].

The site of the bleeding can differ according to the magnitude of the hemorrhage. It can be seen frequently as subinternal limiting membrane. It might also be subhyaloid or a combination of both [2–4]. Vitreous hemorrhage and subretinal hemorrhage have also been described [5].

There are several risk factors that can contribute to this condition. These risk factors may include aerobic exercises [6], weight lifting [7], and balloon inflation [8].

Sexual activity is known to be a risk factor for Valsalva retinopathy. It was first described by Friberg et al. [9] in 1995, followed by several reports [10, 11].

The objective of this report is to describe a case of Valsalva retinopathy after sexual activity in a 54-year-old healthy male.

## 2. Case Report

**Figure 1:**
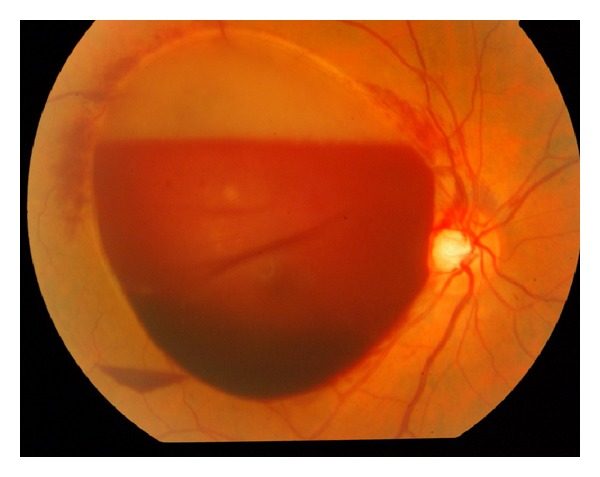
Massive subinternal limiting membrane (ILM) hemorrhage secondary to Valsalva maneuver.

**Figure 2:**
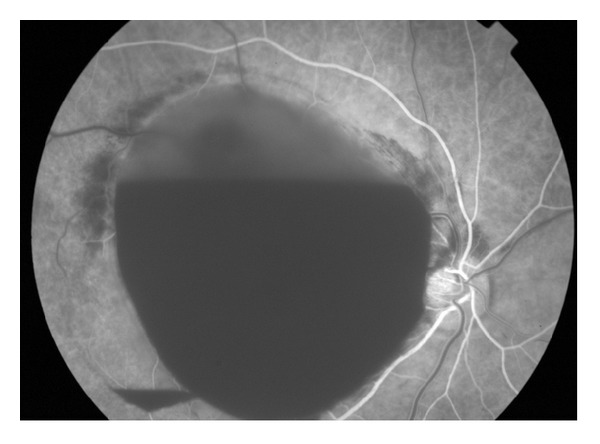
Fluorescein angiography demonstrated hypofluorescence and no evidence of any other pathology.

A 54-year-old healthy man presented to a tertiary referral center complaining of painless decrease of vision in his right eye of four-day duration. The best-corrected visual acuity was counting fingers in the right eye and 20/20 in the left eye. The anterior segment exam was unremarkable. Fundus examination demonstrated a large subinternal limiting membrane (ILM) hemorrhage located in the macular area of the right eye associated with multiple areas of intraretinal hemorrhages (Figure 1). The left eye examination was unremarkable. B-scan ultrasound confirmed a dense diffuse premacular hemorrhage with posterior vitreous detachment. Fluorescein angiography (Figure 2) showed hypofluorescence with no evidence of choroidal neovascular membrane or retinal artery macroaneurysm.

After further questioning about any recent strenuous activities, the patient admitted developing the change in his vision immediately after sexual activity. With this history and examination findings, a diagnosis of Valsalva retinopathy associated with sexual activity was made. No treatment was recommended at that time other than observation for spontaneous recovery.

**Figure 3:**
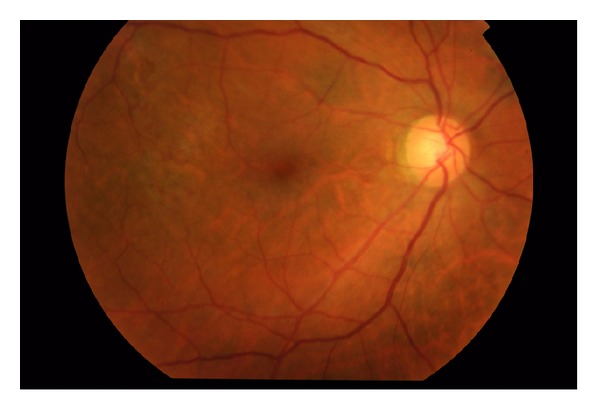
Two weeks after surgery, his best-corrected visual acuity (BCVA) was 20/100 with no hemorrhage and retina was attached.

The patient was followed monthly, and after the third month of followup the visual acuity was still counting fingers, and the hemorrhage had not resolved. The patient was booked for surgical intervention. A 23-gauge sutureless pars plana vitrectomy with brilliant blue assisted internal limiting membrane peeling was performed. Two weeks later, his best-corrected visual acuity (BCVA) was 20/100 with no hemorrhage and retina attached (Figure 3). Six months after surgery, the patient's BCVA was 20/50.

## 3. Discussion

Sexual activity exerts numerous hormonal, hematological, and mechanical alterations on the hemodynamic system of the human body. These alterations represent a risk factor for Valsalva retinopathy regardless of the medical status of the patient. A considerable elevation in intravenous pressure all over the body during Valsalva maneuver increases the possibility for hemorrhages in different places including the retina.

In such conditions and especially if medical history is unclear, it is imperative to exclude any systemic disease that may result in retinal hemorrhages. This can include vascular diseases like diabetes, hypertension, and ocular vein occlusions. Hematological conditions such as sickle cell disease, anemia, coagulopathies, and blood dyscrasias should also be considered.

The first association between sexual activity and retinal hemorrhage was described by Friberg et al. in 1995 [9]. In his series, six cases suffered sudden visual loss associated with sexual activity. The age ranged from 24 to 53 years. Visual acuity in the affected eyes ranged from a mild decrease (20/40) to profound visual loss (counting fingers at 6 in). Fundus exam showed intraretinal, preretinal, or vitreous hemorrhage. Five of the six patients were followed up for at least 1 month and showed spontaneous improvement in vision as the blood cleared. After long-term followup patients enjoyed complete visual recovery without any consequences. Visual prognosis is usually good. Poor visual outcome has been attributed to retinal pigmentary changes at the macula [12].

In 2006, Karagiannis and Gregor [10] reported a case of Valsalva retinopathy following sexual activity in a patient with idiopathic thrombocytopenic purpura and positive antiphospholipid antibodies. This case emphasizes the significance of workup during presentation to exclude any possible treatable condition.

Another series in 2009 was reported by Ahmadabadi et al. [11]. Out of 21 patients, premacular hemorrhage resulted from vigorous sexual activity in 10 patients (47.6%). Among those patients, only one patient was managed with vitrectomy. Neodymium:yttrium-aluminum-garnet (Nd:YAG) laser hyaloidotomy was performed in 8 patients and spontaneous reabsorption was seen in one patient with almost complete visual recovery during followup for all patients.

In the current case, sub-ILM hemorrhage occurred in a 54-year-old healthy male after sexual activity with no significant medical history. After several months of observation, a vitrectomy was performed with good visual outcome.

In summary, sexual activity is a well known risk factor for Valsalva retinopathy. Hemorrhage can be severe and might require surgical intervention. Vitrectomy is an effective and a safe procedure once spontaneous reabsorption does not occur.
